# Automated Image Analysis of HER2 Fluorescence In Situ Hybridization to Refine Definitions of Genetic Heterogeneity in Breast Cancer Tissue

**DOI:** 10.1155/2017/2321916

**Published:** 2017-05-28

**Authors:** Gedmante Radziuviene, Allan Rasmusson, Renaldas Augulis, Daiva Lesciute-Krilaviciene, Aida Laurinaviciene, Eduard Clim, Arvydas Laurinavicius

**Affiliations:** ^1^National Center of Pathology, Affiliate of Vilnius University Hospital Santariskiu Clinics, P. Baublio 5, LT-08406 Vilnius, Lithuania; ^2^Faculty of Natural Sciences, Vilnius University, M. K. Ciurlionio 27, LT-03103 Vilnius, Lithuania; ^3^Faculty of Medicine, Vilnius University, M. K. Ciurlionio 21, LT-03101 Vilnius, Lithuania; ^4^TissueGnostics, Vienna, Austria

## Abstract

Human epidermal growth factor receptor 2 gene- (HER2-) targeted therapy for breast cancer relies primarily on HER2 overexpression established by immunohistochemistry (IHC) with borderline cases being further tested for amplification by fluorescence in situ hybridization (FISH). Manual interpretation of HER2 FISH is based on a limited number of cells and rather complex definitions of equivocal, polysomic, and genetically heterogeneous (GH) cases. Image analysis (IA) can extract high-capacity data and potentially improve HER2 testing in borderline cases. We investigated statistically derived indicators of HER2 heterogeneity in HER2 FISH data obtained by automated IA of 50 IHC borderline (2+) cases of invasive ductal breast carcinoma. Overall, IA significantly underestimated the conventional HER2, CEP17 counts, and HER2/CEP17 ratio; however, it collected more amplified cells in some cases below the lower limit of GH definition by manual procedure. Indicators for amplification, polysomy, and bimodality were extracted by factor analysis and allowed clustering of the tumors into amplified, nonamplified, and equivocal/polysomy categories. The bimodality indicator provided independent cell diversity characteristics for all clusters. Tumors classified as bimodal only partially coincided with the conventional GH heterogeneity category. We conclude that automated high-capacity nonselective tumor cell assay can generate evidence-based HER2 intratumor heterogeneity indicators to refine GH definitions.

## 1. Introduction

Amplification and/or overexpression of the human epidermal growth factor receptor 2 (HER2) oncogene is observed in approximately 20% of invasive breast tumors and is associated with worse prognosis and need for targeted therapy [1–3]. Accurate and precise detection of HER2 status is thus essential for individual therapy decision for patients with breast cancer [4, 5]. The most common procedure for determining HER2 status in breast carcinoma is based on immunohistochemistry (IHC) evaluation of HER2 expression followed by HER2 amplification test in IHC borderline (2+) cases. HER2 amplification is commonly tested by fluorescence in situ hybridization (FISH) detection of HER2 and chromosome 17 (CEP17) FISH signals [6]. Current ASCO/CAP 2013 guidelines [3] state that HER2 amplification must be determined from manual counts of discrete HER2 and CEP17 signals in 40 nuclei per case with an additional 20 nuclei in equivocal cases.

While the majority of HER2 positive and negative tumors can be readily be identified by this procedure, analysis of borderline and heterogeneous tumors may be hampered by cell-to-cell diversity regarding HER2 copy number. HER2 amplified cells may be clustered in specific areas or scattered and intermixed with nonamplified cells [7, 8]. CEP17 polysomy poses another dilemma in interpreting HER2 FISH results; in addition, an increased number of CEP17 signals are related to amplification of the centromeric region rather than to true polysomy [9, 10]. The interpretation of the diversity of cells is further complicated by possible influence of both technical aspects and observer subjectivity in selecting countable nuclei [11–13].

To address the issue of diversity, HER2 genetic heterogeneity (GH) was defined by the College of American Pathologists (CAP) as the presence of more than 5% but less than 50% of infiltrating tumor cells with a HER2/CEP17 ratio >2.2 when using a control probe (or >6 HER2 signals per cell when using a probe for HER2 only) [14]. Seol et al. reported that disease-free survival times in patients with GH were significantly shorter compared with patients without GH [15]. Nevertheless, utility of the GH definition has been questioned by many studies [11–13, 16–18]. In particular, the incidence of GH was reported to vary between 11% and 40% [11], and a higher rate of GH was reported when established by HER2/CEP17 ratio compared to that established by HER2 alone (23% and 7%, resp.) [18]. Furthermore, the GH definition has been shown as noninformative of the underlying distribution within a tumor cell population since the degree of GH increased along with the overall HER2/CEP17 ratio to approach the “cut-off point” of 2.2 [13]. On a similar note, Öhlschlegel et al. reported that the GH was significantly associated with CEP17 polysomy [19].

Manual evaluation of HER2 FISH results is not only a time-consuming and somewhat tedious procedure, but, more importantly, it is based on evaluation of cell diversity in a limited sample and is prone to selection bias [11–13]. The current clinical guidelines for establishing equivocal/heterogeneous cases are complex, involving different quantities and cut-offs (signal counts, their ratio, and proportion of cells amplified) which make them hard to follow. One may argue that the manual assessment of HER2 FISH status is not yet sufficiently standardized in terms of tissue and cell sampling for counting of FISH signals. This was reflected in previous studies of automated FISH evaluation with sampling varying within 20–60 nuclei [20, 21] to a few TMA cores [22] or a few fields selected from breast cancer sections [23].

Automation of HER2 FISH test by means of image analysis (IA) has been proposed by commercial platforms [21–25] to reduce workload and improve precision. While still dependent on good quality samples and standardization of all procedures, IA can aid as decision support tool [21, 23, 26]. Besides the benefits of IA for computer-assisted quantification of FISH signals, significant increases in cell sampling capacity may serve for better assessment of equivocal and heterogeneous cases [20, 21].

Our study explores if objective, statistically derived indicators of HER2 intratumor heterogeneity can be obtained from high-capacity data extracted by IA applied to HER2 FISH digital images.

## 2. Materials and Methods

### 2.1. Patients and Samples

The study included 50 female patients with invasive ductal breast carcinoma diagnosed as borderline HER2 IHC (2+), treated at the National Cancer Institute (Vilnius, Lithuania), and investigated at the National Center of Pathology (Vilnius, Lithuania) between September 2012 and February 2015. The study was approved by the Lithuanian Bioethics Committee.

### 2.2. Fluorescence In Situ Hybridization

4 *μ*m thick sections were stained with PathVysion HER2 DNA Probe Kit (Abbott Molecular, Des Plaines, IL, USA). In this kit, a fluorescently labeled (SpectrumOrange) DNA probe recognizing the HER2 locus (17q11.2-q12) is used in conjunction with a fluorescently labeled (SpectrumGreen) DNA probe recognizing the centromeric region of CEP17 (17p11.1-q11.1). Tissue sections were mounted on positively charged slides, heated overnight at 56°C, deparaffinized in xylene, dehydrated in absolute ethanol, and air-dried. The slides were placed in 0.2 N HCl (pH 0.24) for 20 min, washed in a 2x SSC buffer (pH 7.0), and incubated with pretreatment 1 N NaSCN solution (from Vysis Paraffin Pretreatment Kit, Abbott Molecular, Des Plaines, IL, USA) for 30 min at 80°C. Subsequently, a protease digestion was performed at 37°C for 26 min. The probe mixture was applied to the target tissue and the cover slips were sealed with rubber cement. Denaturation for 5 min at 72°C following hybridization for 19 h at 37°C was performed in a hybridizer (DAKO Diagnostics, Glostrup, Denmark). After hybridization, the slides were washed in 2x SSC/0.3% NP-40 at 72°C for 2 min, air-dried before counterstaining with 4,6-diamidino-2-phenylindole (DAPI) (Invitrogen Corporation, Carlsbad, USA), and covered with a glass coverslip.

### 2.3. Image Acquisition

TissueFAXS-plus (TissueGnostics, Vienna, Austria), a medical device certified for “in vitro” diagnostic (IVD) developed and produced in accordance with ISO13485, was used to scan representative regions of all samples. PanApo 63x/1.4 oil objective (Zeiss, Göttingen) was used for acquiring digital images using a PCO Pixelfly CCD camera. Single band-pass filters are fitted to record nuclei (DAPI), HER2 (Acridine), and CEP17 (FITC) in separately. Each region consisted of a minimum of 4 field of views (FOVs). Image acquisition time of each channel was adjusted to 200 milliseconds.

Digital images were stitched together to regions of interest (ROIs). Each FOV was acquired at 63x magnification and stored at the resolution of 1392 by 1024 pixels, yielding a pixel size of 0.16 *μ*m. To ensure that all signals inside the thick tissue section are available for image analysis, images were acquired using z-stacks composed of 9 steps with a step size of 0,45 *μ*m. Extended depth of focus algorithm of TissueFAXS was used to combine the multiple focal planes. The algorithm is using only the sharpest structures of each layer. An illustration is given in Supplementary Figure  1 (see Supplementary Material available online at https://doi.org/10.1155/2017/2321916).

### 2.4. HER2 FISH Evaluation

Two observers evaluated the mean number of HER2 and CEP17 signals and the HER2/CEP17 ratio per nucleus by conventional manual procedure (MP): 40 nuclei were examined in two or more fields; for equivocal cases, additional 20 nuclei were evaluated. HER2/CEP17 ratios were calculated per tumor by total number of HER2 signals divided by total number of CEP17.

For the automated analysis, ROIs were manually selected for the scanning to ensure good representation and quality of the tumor sample. Automated segmentation of both nuclei and FISH signals was performed with StrataQuest v.205 (TissueGnostics GmbH). All automatically detected nuclei, HER2, and CEP17 signals (automated data (AD)) were reviewed and edited by an observer (GR) on the digital images to produce a set of corrected data (CD) for quality assurance. All nuclei were considered (no filtering of nuclei with less than one of each signal as required by the FISH evaluation guidelines was done before the correction). Subsequently, nuclei without signals or with only one HER2 or CEP17 signal were excluded from further analysis during the statistical analysis.

HER2 amplification status was determined according to the ASCO/CAP guidelines [3] and CEP17 polysomy was defined as an average CEP17 copy number ≥ 3 [27]. The manual analysis utilized MP data, that is, counts within 40–60 nuclei selected by the observers, while the automated analysis used AD data from all extracted nuclei (except the nuclei with insufficient number of FISH signals as indicated above).

The HER2 intratumoral heterogeneity was estimated by (1) the CAP 2009 guidelines, GH, which were applied to both MP and AD to compare the effect of number of included nuclei on the heterogeneity measure and (2) statistical bimodality indicators: Ashman's D and bimodality index. Briefly, bimodality indicators are functions of the parameters describing two Gaussian distributions fitted to the data. The bimodality indicators were calculated for AD distributions of HER2, CEP17, and HER2/CEP17 ratio as extracted per cell by IA.

### 2.5. Statistical Analysis

To assess the accuracy of the AD HER2 copy number, CEP17 copy number and HER2/CEP17 ratio were compared to the CD by paired *t*-test and linear regression. Note that the quality assurance comparison considered all extracted nuclei. Subsequently, the verified AD had the insufficient nuclei (as determined by the ASCO/CAP guidelines) filtered out before comparison to MP. The relationships between signals, ratios, and the bimodality indicators were investigated by factor analysis including results from both AD and MP. On the resulting factor scores, clusters were extracted by the* k*-Means method to explore potential stratification of the cases. Statistical analysis was performed with SAS 9.3 software and *R* 3.1.2. *p* values < 0.05 were considered statistically significant.

## 3. Results

### 3.1. Comparison of Automated and Corrected HER2 FISH Data

Overall, 36,154 nuclei were detected in the digital images from 50 patients. Of those, 27,266 (75.4%) were correctly segmented. 5,626 (15.6%) were under- or oversegmented, and 3,262 (9.0%) were not detected. The mean number of nuclei per tissue section was 723, ranging from 192 to 1,789. The HER2/CEP17 ratios for automated data (AD) and corrected data (CD) were calculated according to the manual procedure (MP) (a sum of HER2 signals divided by sum of CEP17).

Overall, 87,092 HER2 and 65,309 CEP17 signals were detected by the AD. Among them, 81,704 (93.8%) HER2 signals and 1,116 (96.6%) CEP17 signals were correctly detected, while 2,163 (2.5%) and 1,116 (1.7%) were falsely detected, and 3,225 (3.7%) and 1,115 (1.7%) were undetected, respectively.

Paired *t*-test revealed no significant bias between the AD and CD for mean CEP17 copy number (average difference −0.0023, CI = [−0.013; 0.008], *p* = 0.6614) and negligible bias for mean HER2 copy number (average difference 0.046, CI = [0.013; 0.078], *p* = 0.0072) and mean HER2/CEP17 ratio (average difference 0.025, CI = [0.005; 0.0045], *p* = 0.0149). Linear regression analysis showed perfect agreement for all three variables determined by AD and CD (Table 1).

### 3.2. Comparison of Automated and Manual FISH Results

HER2 and CEP17 results obtained by MP and AD (after exclusion of the nuclei with insufficient FISH signals) were compared. Paired *t*-test revealed significantly lower values obtained by AD compared to the MP data: mean HER2 copy number (average difference −1.428, CI = [1.188; 1.668], *p* < 0.0001), mean CEP17 copy number (average difference −0.580, CI = [0.483; 0.676], *p* < 0.0001), and HER2/CEP17 ratio (average difference −0.240, CI = [0.150–0.330], *p* < 0.0001).

Analysis by linear regression analysis (Table 1) confirmed the underestimation bias of HER2, CEP17, and HER2/CEP17 ratio by AD when compared to the MP data. The perfect agreement between the AD and CD shows that there was no significant FISH signal loss during detection by the IA. Yet, one can question if signals could be lost due to quenching or deficiencies from scanning thin focal planes from a thick section. The latter possibility was ruled out since *z*-stacks were acquired and combined to extended focus images; see Supplementary Figure  1. In addition, quality control, comparing the FISH signals by live microscope to the corresponding signals in the scanned images, was performed without any indication of possible signal losses due to signal quenching or scanning process deficiencies.

### 3.3. Impact of Manual and Automated Data on Genetic Heterogeneity Expression

**Figure 1:**
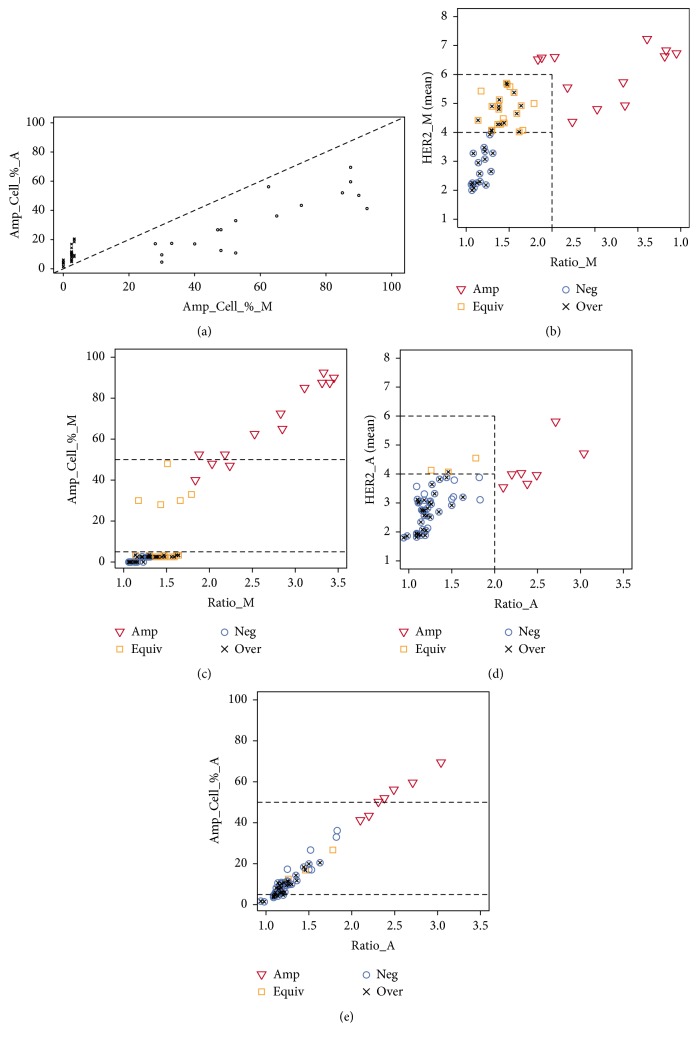
Distribution of tumors with regard to genetic heterogeneity and amplification guidelines. (a) Percentage of amplified cells plotted for MP and AD values, Amp_Cell_%_M and Amp_Cell_%_A, respectively. Dashed line marks identity line. AD overestimates low MP values (crosses) whereas it underestimates MP values in the range >28 (circles); (b) HER2_M plotted against Ratio_M with cut-offs for amplification by ASCO/CAP 2013 guidelines shown by grey lines. Cases marked with crosses are overestimated cases from (a); (c) Cell_Amp_%_M plotted against Ratio_M, horizontal lines at 5% and 50% mark cut-off values for determining GH cases. Note the lack of cases in the 3–28% range; (d) HER2_A plotted against RATIO_A, amplification cut-off marked in grey; (e) Amp_Cell_%_A plotted against RATIO_A. Summary: MP: 13 positive, 21 equivocal, 16 negative, and 8 GH cases; AD: 7 positive, 3 equivocal, 40 negative, and 36 GH cases.

AD underestimated both HER2 and CEP17 counts and HER2/CEP17 ratio per case (Table 1). Similarly, Figure 1(a) shows the percentages of amplified cells were lower in AD compared to MP (Amp_Cell_%_A and Amp_Cell_%_M, resp.) in the range above 25% by MP; however, Amp_Cell_%_A were higher (reaching up to 20%) than Amp_Cell_%_M in the range below 5% by MP.

Figures 1(b) and 1(c) demonstrate how the MP data adhere to the current guidelines for both amplification and GH. The tumors are stratified into nonamplified, equivocal, and amplified (Figure 1(b)); however, a clear gap occurs in the determination of GH cases (Figure 1(c)).  Figure 1(d) reveals the effect of underestimation by AD with fewer cases being amplified or equivocal as they are downgraded into the range for negative cases. The same occurs for the distribution of amplified cell percentages (Amp_Cell_%_A), but, importantly, a continuous distribution of amplified cell percentages can be noted for AD (Figure 1(e)).

Since the current clinical guidelines for amplification and polysomy are defined by the cut-off values for MP data, the AD cannot be readily used for decision support without proper validation. Nevertheless, the more continuous distributions obtained by high-capacity AD may provide more informative measures of cell diversity. One benefit may be related to improved detection of scattered amplified cells which could be missed by MP as it requires tedious screening of large tissue areas. To test this hypothesis, we measured the median distance between the nearest amplified nuclei (with a HER2/CEP17 ratio >2.2) and found significantly (*p* = 0.0138) sparser distribution of amplified nuclei in the AD sets of the 24 potentially heterogeneous cases compared to the 8 cases that were detected as GH by MP (see Supplementary Figure  2). The second benefit of high-capacity analysis is the opportunity to calculate proper objective bimodality indicators; here we focus on bimodality as determined by Ashman's D. To establish a minimal sample size required for reliable detection of bimodality based on Ashman's D criterion, the indicator was calculated on randomly subsampled cells from the AD to simulate incrementally increasing sampling size (from 40 to 1,000 nuclei). With Ashman's D calculated as the mean of 1,000 sampling iterations performed for each sample size, we found that the second peak in the distribution could rarely be detected (in less than half of the subsampling iterations) in a sample size below 200 cells in the tumors (*n* = 23) which were categorized as bimodal based on their full sample size. Accordingly, Ashman's D estimates in this group of tumors revealed improving detection of bimodality (Ashman's D > 2) with increasing sample size where 800 cells potentially would be required for robust detection of this feature (see Supplementary Figure  3). Optimal sampling requirements should be established in larger data sets; however, the simulations clearly show that the required sample size is well beyond the MP capacity.

### 3.4. Factor Analysis

**Figure 2:**
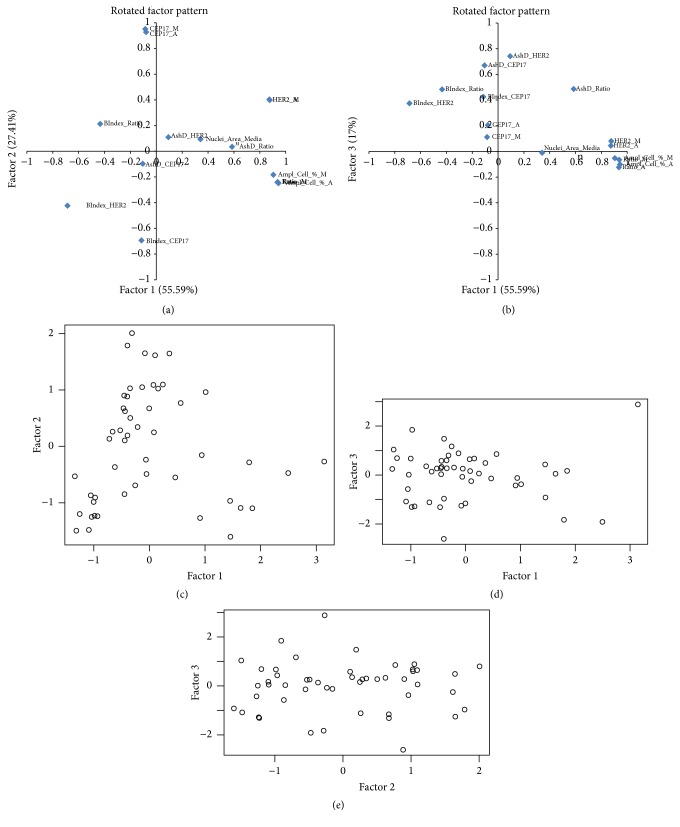
Rotated factor pattern of the indicators obtained by the manual and automated HER2 FISH procedure; *n* = 50. The loadings of (a) factors 1 and 2, (b) factors 1 and 3, and the factor scores (c, d, e) are plotted. Factor 1, amplification; factor 2, polysomy; factor 3, bimodality. Cell_Amp_%_M: percentage of amplified cells detected by manual procedure, calculated from HER2/CEP17 ratio. Cell_Amp_%_A: percentage of amplified cells detected by automated procedure, calculated from HER2/CEP17 ratio. HER2_A, HER2_M - HER2 copy number detected by automated and manual procedures, respectively. CEP17_A, CEP17_M - CEP17 copy number detected by automated and manual procedures, respectively. Ratio_A, Ratio_M - HER2/CEP17 ratio detected by automated and manual procedures, respectively. AshD_Ratio, AshD_HER2, and AshD_CEP17: Ashman's D indicator calculated for HER2/CEP17, HER2, and CEP17 automated data. BIndex_HER2, BIndex_CEP17, and BIndex_Ratio: bimodality indices calculated for HER2, CEP17, and HER2/CEP17 automated data.

The pattern of the 3 factors extracted is presented in Figure 2(a). Factor 1 is characterized by strong positive loadings of the variables indicative of HER2 amplification (including HER2 counts, HER2/CEP17 ratios, and percentages of amplified cells by MP and AD) and is therefore interpreted as the amplification factor. Accordingly, factor 2 can be taken as factor of polysomy. Factor 3 (Figure 2(b)) was characterized by strong positive loadings of the bimodality indicators (mainly from Ashman's D estimated from HER2 and CEP17 distributions, less from the HER2/CEP17 data) and was named the bimodality factor.

### 3.5. Cluster Analysis

**Figure 3:**
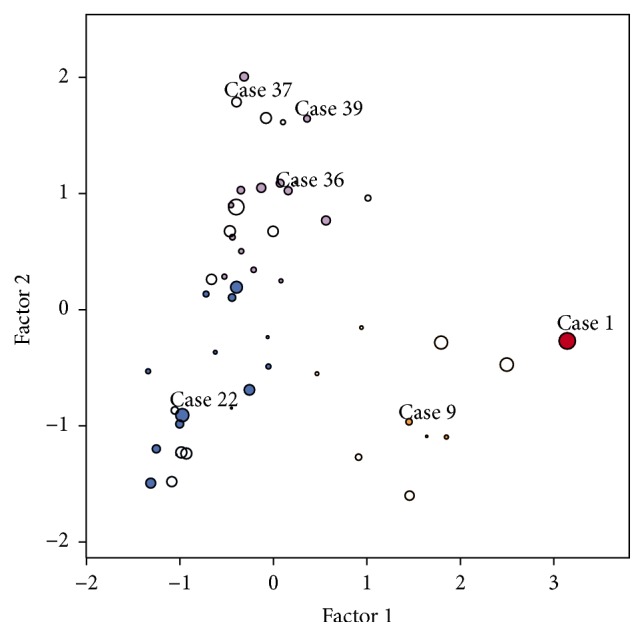
A bubble plot of the clusters obtained from the factor 1, 2, and 3 scores. Cluster colors: Cluster 1, red; Cluster 2, orange; Cluster 3, blue; and Cluster 4, purple. Bubble size represents factor 3 (bimodality); center is empty for negative and filled for positive values. Numbers indicate cluster examples depicted in Figure 4.

While, by definition, the factors are linearly independent, the factor score plots (Figures 2(c)–2(e)) revealed potential nonlinear relationship between the amplification and polysomy factors and clustering of the tumors. A cluster analysis of the 3 factor scores extracted 4 rather distinct clusters presented in Figure 3 and Table 2 (see Table  1 in Supplementary Material for complete listing). The clusters 1 and 2 (containing one and nine cases, resp.) revealed variable degree of amplification and bimodality factor scores. Cluster 3 was represented by nonamplified tumors (18 cases); however, a significant proportion of them revealed high HER2 bimodality score (33.3% cases with Ashman's D > 2). Cluster 4 consisted mainly of equivocal and polysomy cases (22 cases).

**Figure 4:**
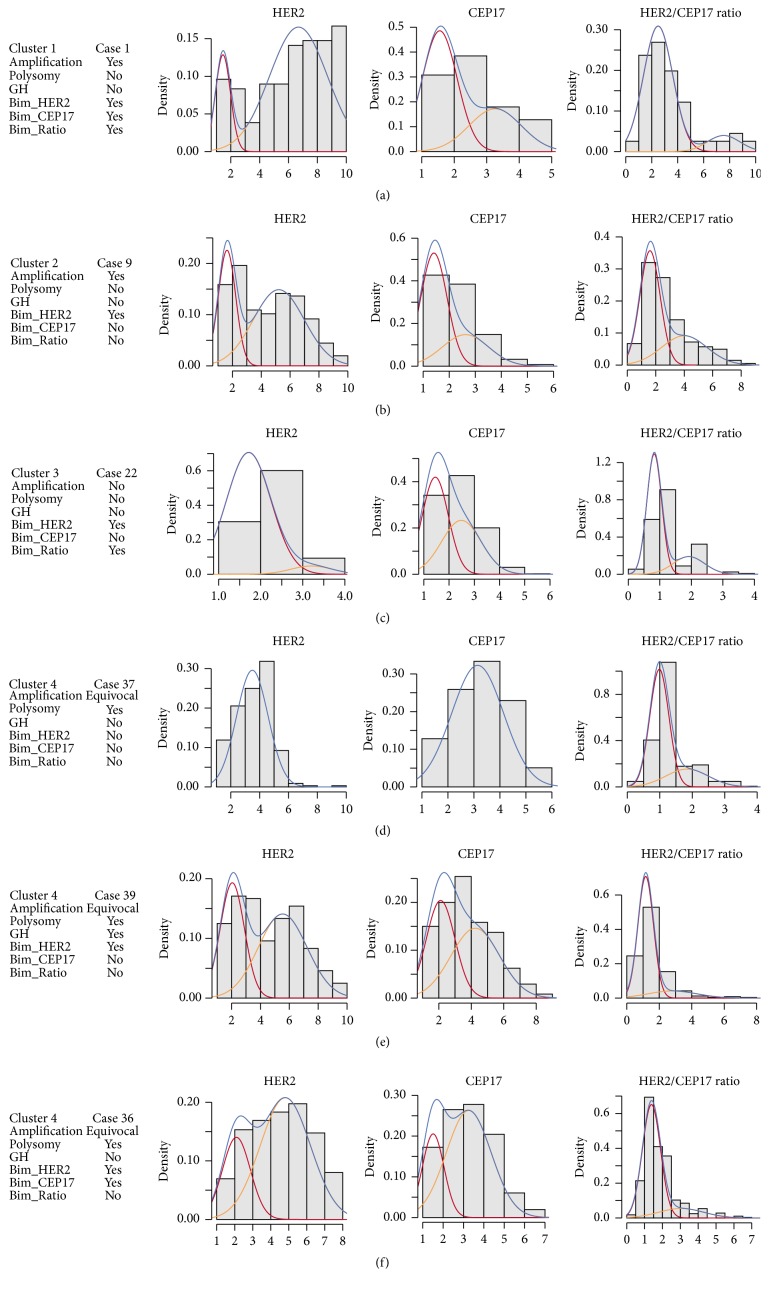
Examples of the tumor cases from the clusters extracted from the automated HER2 FISH data. Histograms of HER2, CEP17, and HER2/CEP17 with Gaussian curves are presented from the cases labeled in the Figure 3. Amplification, polysomy, and genetic heterogeneity categories are based on the conventional manual procedure results. Bim_HER2, Bim_CEP17, and Bim_Ratio represent bimodality categories based on Ashman's D > 2 criterion.

While the cluster analysis distinctly stratified the tumors into amplified, nonamplified, and equivocal/polysomy types, the bimodality factor was variable in all the clusters and provided independent characteristic for the cell diversity. Examples from different clusters are presented in Figure 4. Bimodality of HER2 and/or HER2/CEP17 distribution could be detected as an independent feature in both negative and amplified cases, as in Figures 4(a), 4(b), and 4(c), respectively (none of these cases were categorized as GH by MP). Importantly, the cluster of equivocal and polysomic cases, in Figures 4(d), 4(e), and 4(f), contained some cases with bimodal distribution of HER2 and/or CEP17 and could be categorized as “equivocal with polysomy,” Figure 4(d), and “equivocal with polysomy and HER2 bimodality,” Figures 4(e) and 4(f) cases. Only one case, Figure 4(e), in the example fits the definition of GH.

## 4. Discussion

In our study of 50 HER2 IHC 2+ (borderline) breast carcinomas, we found that automated IA of HER2 FISH images underestimated the conventional assessment by MP and could not readily be used as clinical decision support tool to measure the level of HER2 amplification. However, the benefit of high-capacity nonselective tumor cell assay could be utilized to generate unbiased, quantitative indicators of HER2 intratumor heterogeneity. Importantly, AD revealed a more continuous distribution of the fraction of amplified cells compared to the MP results. Furthermore, AD enabled characteristics of intratumor heterogeneity based on bimodality indicators rather than the fraction of amplified cells. The method also allowed extraction of linearly independent scores of amplification, polysomy, and bimodality with subsequent stratification into relatively unimodal and bimodal tumors; the latter category only partially overlapped with the conventional GH cases.

Clinical HER2 FISH testing is rather straightforward in nonamplified and amplified cases; however, it presents a serious challenge in evaluation of equivocal cases, both by conventional and image analysis-based methods [21, 23, 28]. When therapy decision is to be based on variations within a rather limited number of cells and somewhat arbitrary definitions and cut-off values, robust and evidence-based approaches are needed. Rather than verifying or merely assisting the manual counts of a limited number of cells, high-capacity IA can provide an added value to overcome the limitations of conventional HER2 testing, in particular, by refining the GH concept and highlighting the impact of CEP17 variability on proper interpretation of the test results.

We demonstrate that mathematical bimodality indicators retrieved from the AD are linearly independent of the level of HER2 amplification or CEP17 polysomy (or HER2/CEP17 ratio). They can therefore serve as objective and quantifiable measures of intratumor heterogeneity, based on true intratumor variation. This is superior to the GH concept which is largely based on the fraction of amplified cells as previously demonstrated by Chang et al. [13] to be dependent on the overall level of amplification. This association was also noted in our study (Figure 1(c)). Bimodality indicators reflect the distribution pattern of the cell population tested and therefore convey different characteristics of the tumor heterogeneity compared to the GH concept. Importantly, we found only partial overlap between GH and bimodal (Ashman's D > 2) cases in our study. Furthermore, in our experiments with random simulations of different sample size, we observed that robust detection of the bimodality feature of HER2 amplification in breast cancer tissue requires at least 800 cells. Although larger data sets are needed to establish the cell sampling requirements, they are obviously beyond the MP capacity.

High-capacity automated IA presents another potential benefit for HER2 FISH testing as it sheds the light into distribution of unselected cells and may detect rare amplified cells dispersed in a larger cell population. Comparison of MP and AD in our study with regard to the GH definitions revealed peculiar differences in the amplified cell distribution. Amp_Cell_%_A and Amp_Cell_%_M plots (Figure 1) exposed a large distribution gap in the MP data. Namely, all GH cases detected by MP contained at least 28% amplified cells. On the other hand, out of 32 cases with Amp_Cell_%_M under 5%, 24 cases disclosed 5 to 21% by Amp_Cell_%_A (there were no cases detected by MP in the interval from 5 to 21%). There were 9 cases with less than 5% amplified cells detected automatically. This discrepancy might be associated with scattered amplified cells which could be missed by MP as it requires tedious screening of large tissue area. To support this hypothesis, we found that the median distance between the amplified nuclei was significantly higher in the cases undisclosed by MP. It points to the potential of IA to capture the cell amplification/heterogeneity to full extent. The range of the gap fits well with reports from other studies investigating the amplification cut-off values in the GH guidelines: the questionability of the lower limit (5%) of the amplification threshold was argued by a possible misclassification of the case as heterogeneous when only 1 cell with a HER2/CEP17 ratio >2.2 in 20 is counted [12, 16, 18]. Layfield and Schmidt showed that the cells with 3 : 1 HER2/CEP17 ratio were the determining factor for GH in 46% of heterogeneous cases, while 35% of GH cases were established due to a single 3 : 1 cell [16]. It is known that loss of CEP17 signal may be the result of nuclear truncation [29]. On the other hand, Bartlett et al. reported that only cases containing >30% of amplified cells (HER2/CEP17 ratios more than 2.2) were associated with lower disease-free survival [12]. Allison et al. showed that different amplification ranges dichotomize cohorts very differently: nonamplified and heterogeneous cases accumulated in the 5% to 15% range, while equivocal and heterogeneous gathered in the 25% to 35% interval [18]. They suggested that the recommended thresholds used for reporting heterogeneity may be too low.

Our study is limited by the lack of outcome data to test the clinical/predictive value of the bimodality indicators for evaluation of HER2 amplification intratumor heterogeneity. Neither was it our goal to calibrate a computer-assisted tool to count FISH signals in a limited number of cells according to the current clinical guidelines. Previous studies on HER2 FISH image analysis to support evaluation of HER2 status did not explore the advantages of larger cell sampling and were usually based on 40–60 nuclei [22, 23], or “at least 60 valid nuclei” [21], or “an average of 113 cells per case (median 94, range 47–254)” [25]. Our results suggest that automated image analysis can add value by retrieving new quality information on intratumor heterogeneity rather than merely supporting manual evaluation according to the current clinical guidelines. Indeed, bimodality indicators are more mathematically appropriate and may prove to be more biologically relevant features of intratumor heterogeneity to be considered in clinical settings and definitions of GH. Of note, bimodality indicators of Ki67 IHC data have been recently shown to be an independent prognostic factor of overall survival in breast cancer patients; remarkably, the bimodality of intratumor distribution of Ki67 positive tumor cells was more powerful prognostic factor than the Ki67 labeling index per se [30].

In conclusion, the automated HER2 FISH image analysis in our study underestimated the HER2 and CEP17 data obtained by conventional HER2 FISH test. This bias is most likely caused by cell selection differences in the manual and automated procedures and makes the image analysis not readily applicable according to the current clinical guidelines. However, a unique benefit of the automated high-capacity nonselective tumor cell assay can be obtained from generating unbiased, quantitative indicators of HER2 intratumor heterogeneity with regard to HER2, CEP17 signals, and their ratios. The method also allowed extraction of linearly independent scores of amplification, polysomy, and bimodality with subsequent stratification into relatively unimodal and bimodal tumors, with only partial overlap to the conventional GH cases. Importantly, IA revealed a continuous distribution of amplified cells without gaps which improved detection of rare amplified cells in the tumors with less than 5% amplified cells by conventional HER2 FISH test.

## Supplementary Material

Supplementary Figure 1. Image Acquisition Left: Extended focal plane x-y image of a cell containing multiple HER2 and CEP17 signals. The horizontal lines indicate orthogonal visualization planes. Right: The three planes visualized along x-z axis through the acquired z-stack. It is seen that the dots located inside the tissue are captured within the range of z-stack acquisition. Supplementary Figure 2. Distribution of log-transformed median distance between nearest amplified nuclei in heterogeneous tumors by AD and MP. Group A (*n* = 24) contains potentially heterogeneous cases by AD (5≤Ampl_Cell_%_A<25), which were not detected as heterogeneous by MP. Group B (*n* = 8) represents heterogeneous cases by MP (5≤Ampl_Cell_%_M<50). The log-transformed median distance was higher in the group A (2.4024) compared to the group B (2.2249), *p* = 0.0138. Supplementary Figure 3. Distribution of Ashmans'D values based on random cell sample size simulations. Horizontal axis represents the sample size of randomly selected cells. Vertical axis represents Ashman's D values. Box and whisker plots of the of mean Ashman's D values obtained from randomly subsampled cell populations of the AD set are presented for the two groups based on their full sample Ashman's D value: group A includes cases with Ashman's D > 2 (*n* = 23), group B – Ashman's D ≤ 2 (*n* = 27). Supplementary Table 1. Cluster Summary. Complete listing of amplification state, genetic heterogeneity (GH), polysomy and bimodality, AshD_Ratio, AshD_Her2, AshD_CEP17 – Ashman's D indicator calculated for Her2/CEP17, Her2, and CEP17 automated data.

## Figures and Tables

**Table 1:** Regression analysis of HER2 copy number, CEP17 copy number, and HER2/CEP17 ratio. Automated data (AD) was tested as dependent variable to estimate its prediction from the corrected data (CD) and manual procedure (MP) results.

Variable	*R*2	Intercept	Intercept *p*	Slope	Slope *p*
AD dependent, CD explanatory					
HER2 copy number	0.989	0.209	*p* < 0.0001	0.918	*p* < 0.0001
CEP17 copy number	0.992	0.073	*p* = 0.0106	0.968	*p* < 0.0001
HER2/CEP17 ratio	0.985	0.099	*p* = 0.0002	0.915	*p* < 0.0001
AD dependent, MP explanatory					
HER2 copy number	0.851	0.610	*p* = 0.0004	0.557	*p* < 0.0001
CEP17 copy number	0.84	0.464	*p* < 0.0001	0.621	*p* < 0.0001
HER2/CEP17 ratio	0.92	0.335	*p* < 0.0001	0.784	*p* < 0.0001

**Table 2:** Characteristics of the clusters extracted from the automated image analysis data. GH by Cell_Amp_%_M: percentage of amplified cells detected by manual procedure, calculated from HER2/CEP17 ratio and by HER2 signal only. AshD_Ratio, AshD_HER2, and AshD_CEP17: Ashman's D indicator calculated for HER2/CEP17, HER2, and CEP17 automated data.

	Cluster 1	Cluster 2	Cluster 3	Cluster 4	Total
Number of observations	1	9	18	22	50
Amplification (amp/equiv/neg)	1/0/0	9/0/0	0/4/14	3/17/2	50
Polysomy	0	0	3	19	22
GH by Cell_Amp_%_M (by HER2/CEP17 ratio)	0	1	1	6	8
GH by Cell_Amp_%_M (by HER2 only)	0	4	6	17	27
AshD_Ratio > 2	1	1	2	1	5
AshD_Her2 > 2	1	4	6	12	23
AshD_CEP17 > 2	1	0	5	5	11
Predominantly	Amplified	Amplified	Negative	Equivocal Polysomic Bimodal	
