# Supplementary material for: Automated Image Analysis of HER2 Fluorescence In Situ Hybridization to Refine Definitions of Genetic Heterogeneity in Breast Cancer Tissue
Source: Biomed Res Int. 2017 May 28;2017:2321916. doi: 10.1155/2017/2321916 (PMC5511668; doi:10.1155/2017/2321916)
Supplement: Supplementary file 1 — Supplementary Figure 1. Image Acquisition Left: Extended focal plane x-y image of a cell containing multiple HER2 and CEP17 signals. The horizontal lines indicate orthogonal visualization planes. Right: The three planes visualized along x-z axis through the acquired z-stack. It is seen that the dots located inside the tissue are captured within the range of z-stack acquisition. Supplementary Figure 2. Distribution of log-transformed median distance between nearest amplified nuclei in heterogeneous tumors by AD and MP. Group A (n = 24) contains potentially heterogeneous cases by AD (5≤Ampl_Cell_%_A<25), which were not detected as heterogeneous by MP. Group B (n = 8) represents heterogeneous cases by MP (5≤Ampl_Cell_%_M<50). The log-transformed median distance was higher in the group A (2.4024) compared to the group B (2.2249), p = 0.0138. Supplementary Figure 3. Distribution of Ashmans'D values based on random cell sample size simulations. Horizontal axis represents the sample size of randomly selected cells. Vertical axis represents Ashman's D values. Box and whisker plots of the of mean Ashman's D values obtained from randomly subsampled cell populations of the AD set are presented for the two groups based on their full sample Ashman's D value: group A includes cases with Ashman's D > 2 (n = 23), group B – Ashman's D ≤ 2 (n = 27). Supplementary Table 1. Cluster Summary. Complete listing of amplification state, genetic heterogeneity (GH), polysomy and bimodality, AshD_Ratio, AshD_Her2, AshD_CEP17 – Ashman's D indicator calculated for Her2/CEP17, Her2, and CEP17 automated data. [file 2321916.f1.docx]

# Supplementary Material


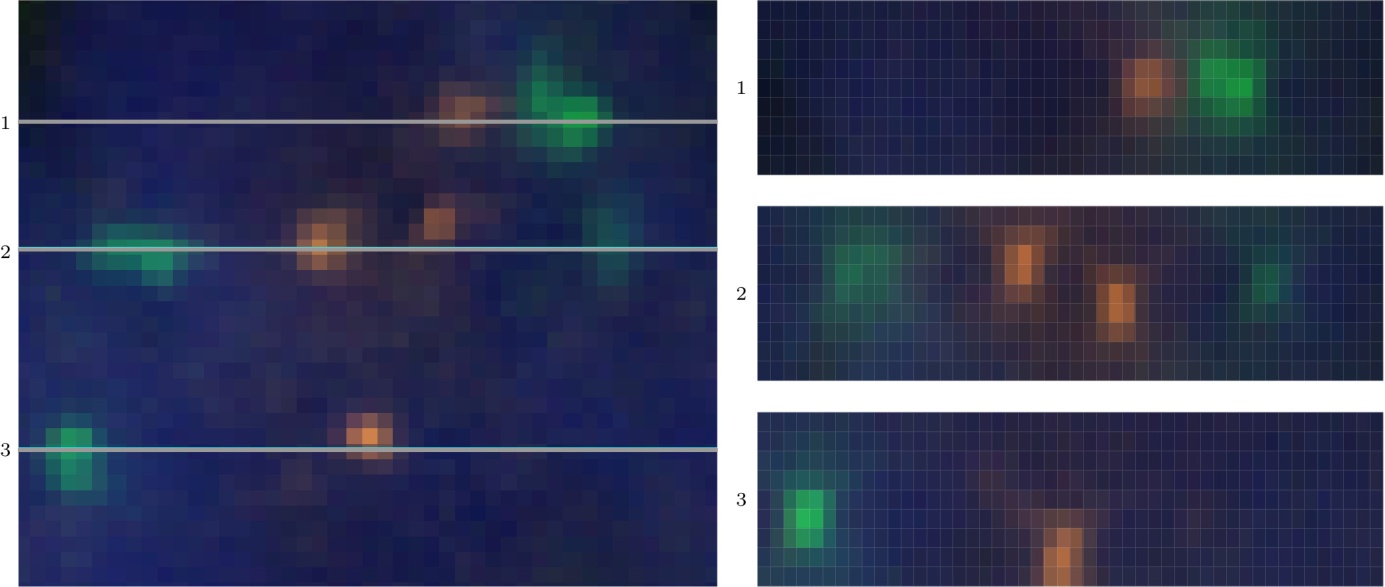


Supplementary Figure 1. Image Acquisition

Left: Extended focal plane x-y image of a cell containing multiple HER2 and CEP17 signals. The horizontal lines indicate orthogonal visualization planes. Right: The three planes visualized along x-z axis through the acquired z-stack. It is seen that the dots located inside the tissue are captured within the range of z-stack acquisition.


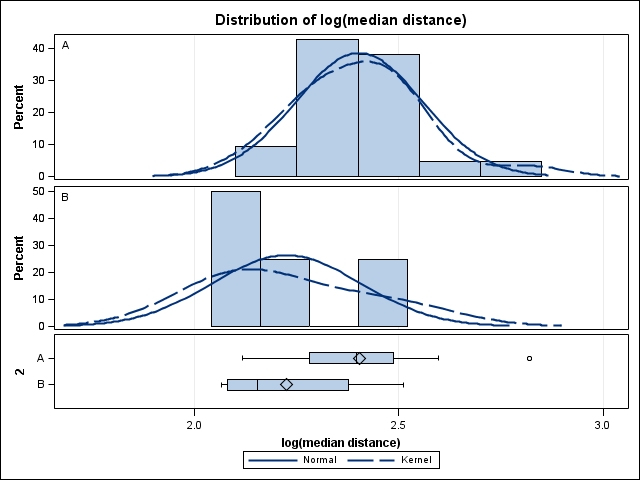


**Supplementary Figure 2. Log-transformed median distance between nearest amplified nuclei in heterogeneous tumors by AD and MP.**

Group A (n=24) contains potentially heterogeneous cases by AD (5≤Ampl_Cell_%_A<25), which were not detected as heterogeneous by MP. Group B (n=8) represents heterogeneous cases by MP (5≤Ampl_Cell_%_M<50). The log-transformed median distance was higher in the group A (2.4024) compared to the group B (2.2249), p = 0.0138.


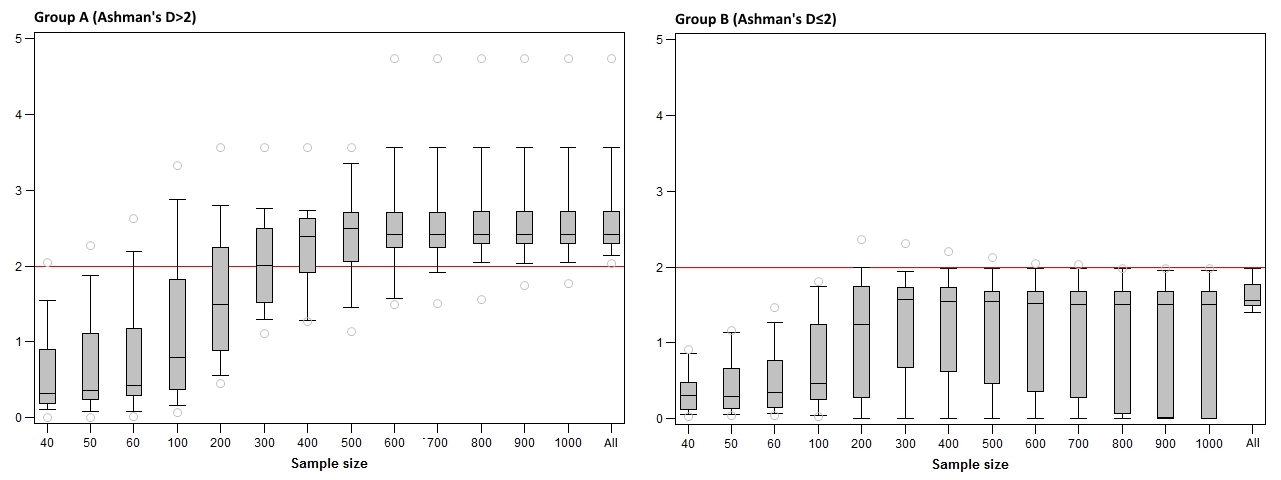


Supplementary Figure 3. Distribution of Ashmans’D values based on random cell sample size simulations.

Horizontal axis represents the sample size of randomly selected cells. Vertical axis represents Ashman’s D values. Box and whisker plots of the of mean Ashman’s D values obtained from randomly subsampled cell populations of the AD set are presented for the two groups based on their full sample Ashman’s D value: group A includes cases with Ashman’s D>2 (n=23), group B – Ashman’s D≤2 (n=27).

| Cluster | ID | Amplification | GH | Polysomic | Bim_HER2 | Bim_CEP17 | Bim_Ratio |
| --- | --- | --- | --- | --- | --- | --- | --- |
| 1 | 1 | Amplified | No | No | Yes | Yes | Yes |
| 2 | 2 | Amplified | No | No | No | No | No |
| 2 | 3 | Amplified | No | No | No | No | No |
| 2 | 4 | Amplified | No | No | No | No | No |
| 2 | 5 | Amplified | No | No | No | No | No |
| 2 | 6 | Amplified | No | No | Yes | No | No |
| 2 | 23 | Amplified | Yes | No | No | No | Yes |
| 2 | 7 | Amplified | No | No | Yes | No | No |
| 2 | 8 | Amplified | No | No | Yes | No | No |
| 2 | 9 | Amplified | No | No | Yes | No | No |
| 3 | 10 | Negative | No | No | No | Yes | No |
| 3 | 11 | Negative | No | No | No | No | No |
| 3 | 12 | Negative | No | No | Yes | No | No |
| 3 | 35 | Equivocal | No | Yes | Yes | No | No |
| 3 | 13 | Negative | No | No | No | No | No |
| 3 | 14 | Negative | No | No | Yes | No | No |
| 3 | 15 | Equivocal | No | No | Yes | No | No |
| 3 | 16 | Negative | No | No | No | No | Yes |
| 3 | 17 | Negative | No | No | No | No | No |
| 3 | 18 | Negative | No | No | No | Yes | No |
| 3 | 19 | Negative | No | No | No | Yes | No |
| 3 | 20 | Negative | No | No | No | No | No |
| 3 | 21 | Negative | No | No | No | No | No |
| 3 | 22 | Negative | No | No | Yes | No | Yes |
| 3 | 46 | Negative | No | Yes | Yes | Yes | No |
| 3 | 24 | Negative | No | No | No | No | No |
| 3 | 26 | Equivocal | Yes | No | No | No | No |
| 3 | 27 | Equivocal | No | No | No | Yes | No |
| 4 | 28 | Amplified | Yes | Yes | Yes | Yes | No |
| 4 | 29 | Equivocal | No | Yes | Yes | No | No |
| 4 | 30 | Amplified | Yes | Yes | No | Yes | No |
| 4 | 31 | Equivocal | No | Yes | No | No | No |
| 4 | 32 | Equivocal | Yes | Yes | No | No | No |
| 4 | 33 | Equivocal | No | No | Yes | No | No |
| 4 | 34 | Equivocal | No | Yes | Yes | No | No |
| 4 | 36 | Equivocal | No | Yes | Yes | Yes | No |
| 4 | 37 | Equivocal | No | Yes | No | No | No |
| 4 | 38 | Equivocal | No | No | Yes | No | Yes |
| 4 | 39 | Equivocal | Yes | Yes | Yes | No | No |
| 4 | 40 | Equivocal | No | Yes | No | No | No |
| 4 | 41 | Equivocal | Yes | Yes | Yes | Yes | No |
| 4 | 42 | Equivocal | No | Yes | Yes | No | No |
| 4 | 43 | Equivocal | Yes | No | Yes | No | No |
| 4 | 44 | Equivocal | No | Yes | Yes | Yes | No |
| 4 | 45 | Equivocal | No | Yes | No | No | No |
| 4 | 47 | Equivocal | No | Yes | No | No | No |
| 4 | 48 | Negative | No | Yes | No | No | No |
| 4 | 49 | Amplified | No | Yes | No | No | No |
| 4 | 25 | Negative | No | No | No | No | No |
| 4 | 50 | Equivocal | No | Yes | Yes | No | No |

**Supplementary Table 1.** Cluster Summary.

Complete listing of amplification state, genetic heterogeneity (GH), polysomy and bimodality, AshD_Ratio, AshD_Her2, AshD_CEP17 – Ashman’s D indicator calculated for Her2/CEP17, Her2, and CEP17 automated data.
